# Editorial: Exploring neuroinflammatory pathways that contribute to chronic pain

**DOI:** 10.3389/fphar.2022.1025200

**Published:** 2022-09-15

**Authors:** Lisa A. Lione, Amy Fisher

**Affiliations:** ^1^ University of Hertfordshire, Hatfield, United Kingdom; ^2^ GW Pharmaceuticals, Cambridge, United Kingdom

**Keywords:** neuroinflammation, chronic pain, ferroptosis, protease activated receptors, SIRT2, *Panax ginseng* (C. A. Meyer)

Chronic pain leading to a reduced quality of life is one of the most important health problems worldwide. It has been estimated that 10% of adults are diagnosed with chronic pain each year. However, despite the high prevalence of chronic pain, its treatment options are limited, in part, due to the variety of chronic pain conditions with different aetiologies (e.g., diabetes, cancer, viral, musculoskeletal) and because their pathophysiological mechanisms are only partially known and can change with time. Therefore, there is a huge unmet need for new effective therapies for the control and/or prevention of multifarious chronic pain conditions.

Chronic pain is associated with neuroinflammation where cellular and molecular immune components such as microglia and astrocytes, cytokines, complement, and pattern-recognition receptors act as key regulators of pain signalling. Emerging evidence indicates long-term effects of COVID-19 infection can exacerbate neuroinflammation conditions underlying chronic pain. The exact mechanisms underlying the link between neuroinflammation, and chronic pain are still not clear and the study of innovative approaches targeting neuroinflammation pathways and their resolution is currently an emerging field of pain research.

The goal of this Research Topic is to compile a series of articles focused on identifying, treating, and modulating neuroinflammation-associated chronic pain, aiming to improve our understanding and enhance the development of novel therapeutic strategies.

The immune system plays a fundamental role in the generation of inflammation which is a protective reaction of the body against injury or infection. Dysregulation of the immune system can lead to conditions such as cytokine storm which can be life threatening. Protease activated receptors (PARs) play an important role in mediating some of the inflammatory and pain responses associated with immune-mediated inflammatory conditions for example Osteoarthritis (OA). O’Brien and McDougall demonstrated that the role of PAR4 may be engaged early in OA disease progression i.e., during the acute predominantly inflammatory phase, but not late phase. Blockade of PAR4 may therefore be most suitable for early OA pain treatment or during episodic flares. Chronic inflammation and associated pain can also be caused by oxidative stress during ferroptosis, a process of apoptotic lipid peroxidation caused by iron overload. SIRT2 (a NAD + - dependent deacetylase) may regulate oxidative stress (ref in Zhang et al.) and increasing evidence indicates that SIRT2 is involved in inflammatory processes particularly prominent in e.g. arthritis (references in Sun et al., 2020). In this Research Topic Zhang et al. presented the first data that SIRT2 relieves mechanical allodynia, enhances ferroportin 1, inhibits intracellular iron accumulation and reduces oxidant stress levels in a rat spared nerve injury (SNI)-induced neuropathic pain model. Two other studies support this view (Zhang and Chi, 2018; Guo et al., 2021) consequently, understanding the physiological mechanism of SIRT2 may help inform clinical treatment of neuropathic pain.

The current Research Topic also includes critical reviews and a perspective paper. Johnston et al. review how the interaction between the immune system and the nervous system contribute to the development and maintenance of chronic pain in production animals (e.g., sheep, cattle, and pigs) highlighting important gaps in our knowledge of the pathophysiology and measurement of pain states in livestock. Livestock offer promising translational models of naturally occurring painful conditions in humans (Herzberg and Bustamante, 2021) due to their larger size (compared to typical rodent models), similar anatomy and histomorphology of regions of interest to human neuroimmune and pain processing (Alvites et al., 2021). The development and application of objective measures of pain *per se* e.g., biomarker tests in production animals (like in rodents and humans, Kwok et al., 2013) present exciting opportunities to improve our fundamental understanding of chronic pain and better inform human clinical pain management and animal husbandry practices and pain interventions. Gada et al. review and emphasise the importance of identifying signature miRNA regulatory networks (small non-coding RNA involved in human diseases) in neuroinflammation and associated neuropathic pain with a focus on human and rodent microRNAs. This they suggest, will help facilitate the discovery of novel miRNA/target biomarkers for more effective diagnosis, prognosis, and management of neuropathic pain in the future. Li et al. provide a mini review for the theoretical basis (regulation of pro-inflammatory cytokine expression and modulation of estrogen receptors) and clinical treatment of multiple types of chronic pain (neuropathic, inflammatory, osteoarthritis and abdominal) using the traditional oriental herbal drug *Panax ginseng* (ginseng and its active constituents including ginsenosides). Finally, Valentine et al. emphasize an important link between vascular-immune interactions and chemotherapy-induced proinflammatory neuropathic pain. This perspective highlights the fundamental importance of the permeability of the vasculature within the somatosensory nervous system in promoting a pro-inflammatory environment and the development of neuropathic pain.

In conclusion, the Research Topic explores an array of neuroinflammatory mechanisms from serine proteases in early OA, ferroptosis in NP, miRNA regulatory networks and vascular-immune interactions linked to chronic pain. Evidence has been presented for innovative therapeutic target approaches for chronic pain, including *Panex Ginseng*, PAR4 and SIRT2. However, as reflected by the original research papers and reviews, it is clear that more research is needed to identify central neuroinflammatory mechanisms driving chronic pain as well as the identification of reliable and translational objective biomarkers of pain in both the preclinical and clinical setting.

